# Supplementary material for: Mechanically robust neuroprotective stent by sequential Mg ions release for ischemic stroke therapy
Source: Nat Commun. 2025 Jul 16;16:6557. doi: 10.1038/s41467-025-61199-x (PMC12267439; doi:10.1038/s41467-025-61199-x)
Supplement: Supplementary file 1 — Supplementary Information [file 41467_2025_61199_MOESM1_ESM.pdf]

Supplementary Materials for

Mechanically robust neuroprotective stent by sequential Mg ions release for ischemic stroke therapy

Hongkang Zhang *et al.*

* Corresponding author: jixm@ccmu.edu.cn (Xunming Ji); jiangmiaowen415@163.com (Miaowen Jiang); liming@xwhosp.org (Ming Li); yfzheng@pku.edu.cn (Yufeng Zheng).

.

This PDF file includes:

Fig. S1 to S25

Table S1 to S4

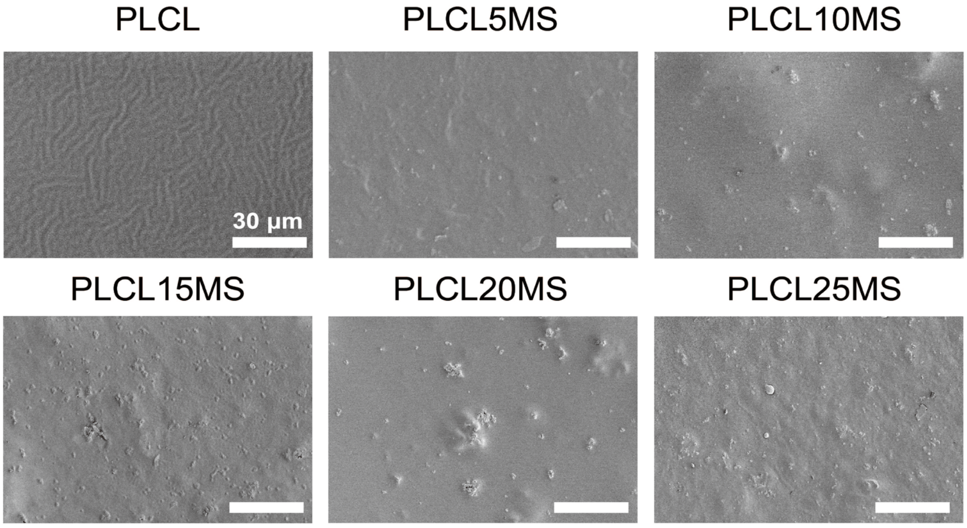

Fig. S1.

SEM image of PLCLxMS (x=0, 5, 10, 15, 20, 25) sheets.

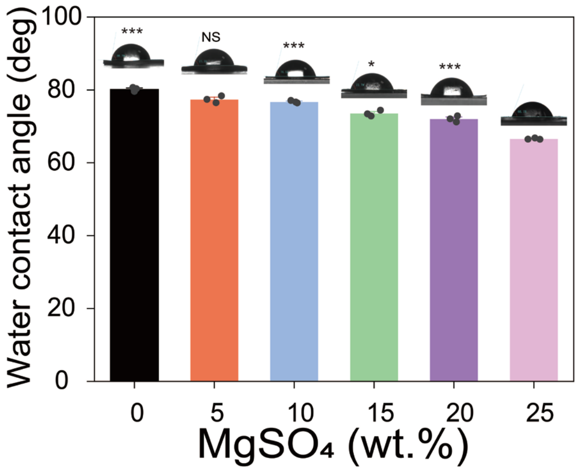

Fig. S2.

Contact angle test of PLCLxMS (x=0, 5, 10, 15, 20, 25) sheets. Sample size: n = 3 biologically independent replicates. * $P < 0.05$, PLCL15MS vs. PLCL20MS group; *** $P < 0.001$, PLCL vs. PLCL5MS group or PLCL10MS vs. PLCL15MS group or PLCL20MS vs. PLCL25MS group; NS, not significant, PLCL5MS vs. PLCL10MS group. Source data and exact P values are provided as a Source data file. One-way analysis of variance (ANOVA) with a Tukey/Games-Howell post hoc test for multiple comparisons. Data are presented as mean values ± SD.

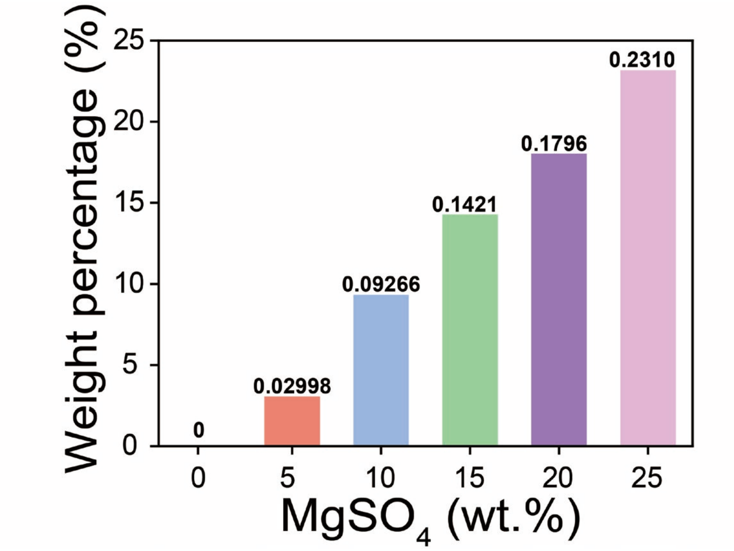

Fig. S3.

Precise content of corresponding magnesium sulfate in different composites from DSA. Source data are provided as a Source Data file.

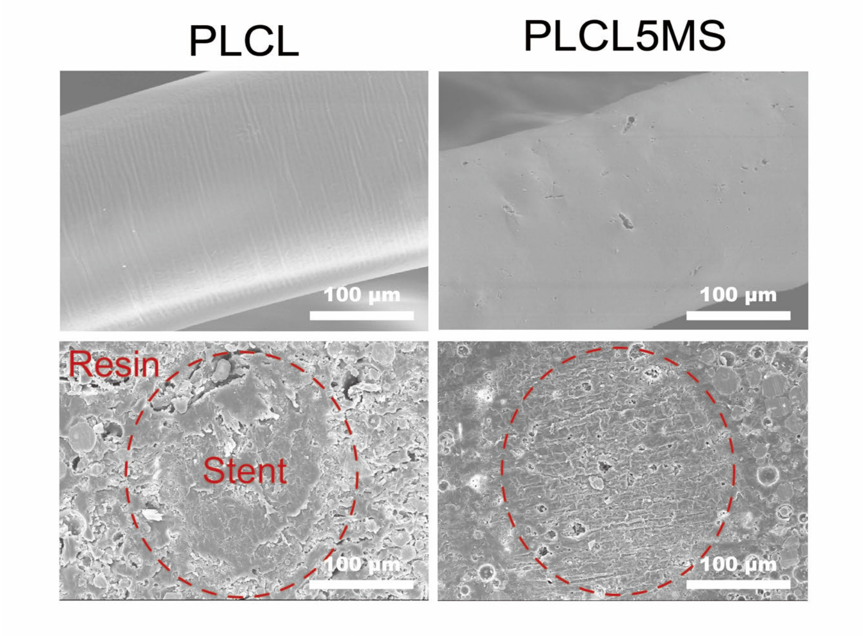

Fig. S4.

Surface and cross-sectional morphology of the PLCL and PLCL10MS stents after immersion in PBS for 7 days.

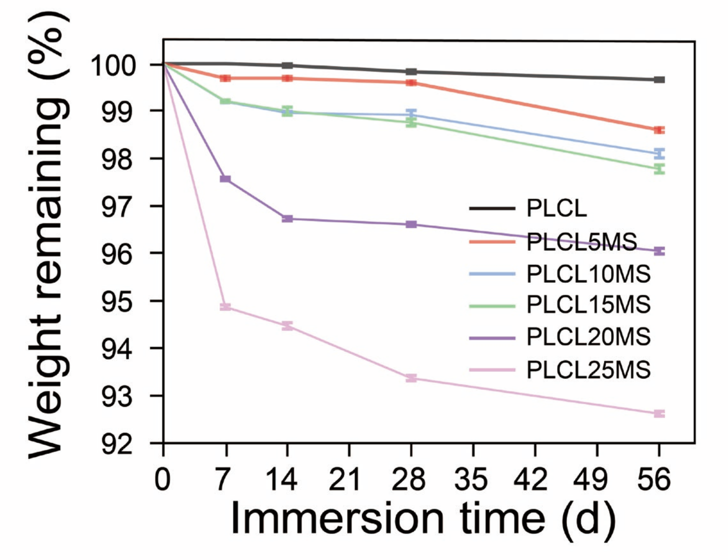

Fig. S5.

Weight loss curve of PLCLxMS stents within 56 days of immersion in PBS. Source data and exact P values are provided as a Source data file. One-way analysis of variance (ANOVA) with a Tukey/Games-Howell post hoc test for multiple comparisons. Sample size: n=3 biologically independent replicates. Data are presented as mean values ± SD.

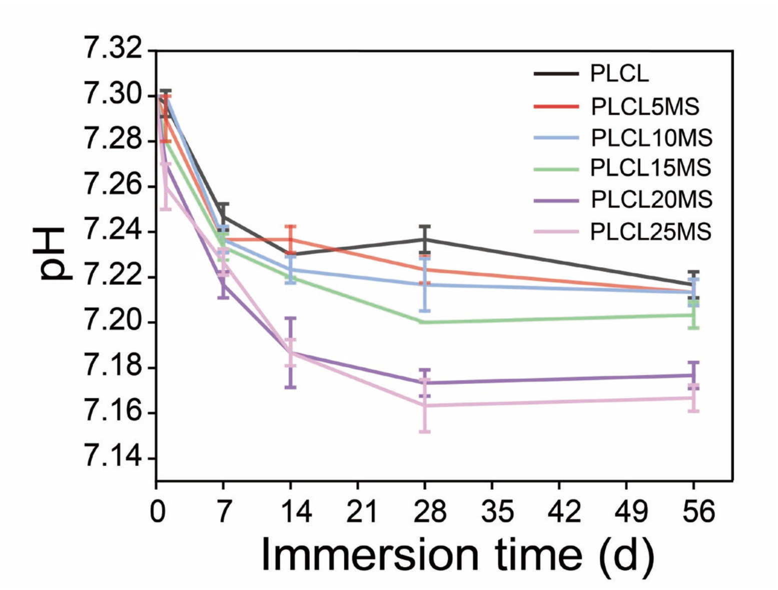

Fig. S6.

The pH change of PBS within 56 days of immersion of PLCLxMS (x=0, 5, 10, 15, 20, 25) stents. Source data and exact P values are provided as a Source data file. One-way analysis of variance (ANOVA) with a Tukey/Games-Howell post hoc test for multiple comparisons. Sample size: n=3 biologically independent replicates. Data are presented as mean values ± SD.

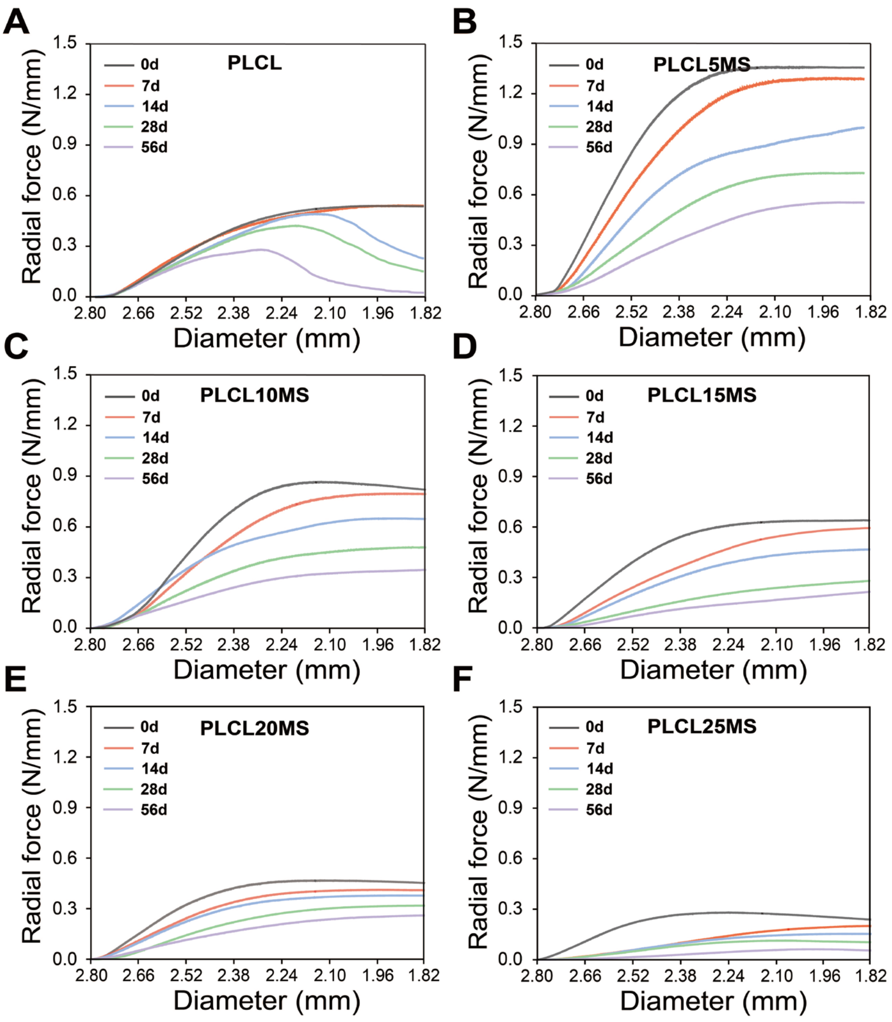

Fig. S7.

Radial force of PLCLxMS (x=0, 5, 10, 15, 20, 25) stents measured after immersion for 0, 7, 14, 28 and 56 days.

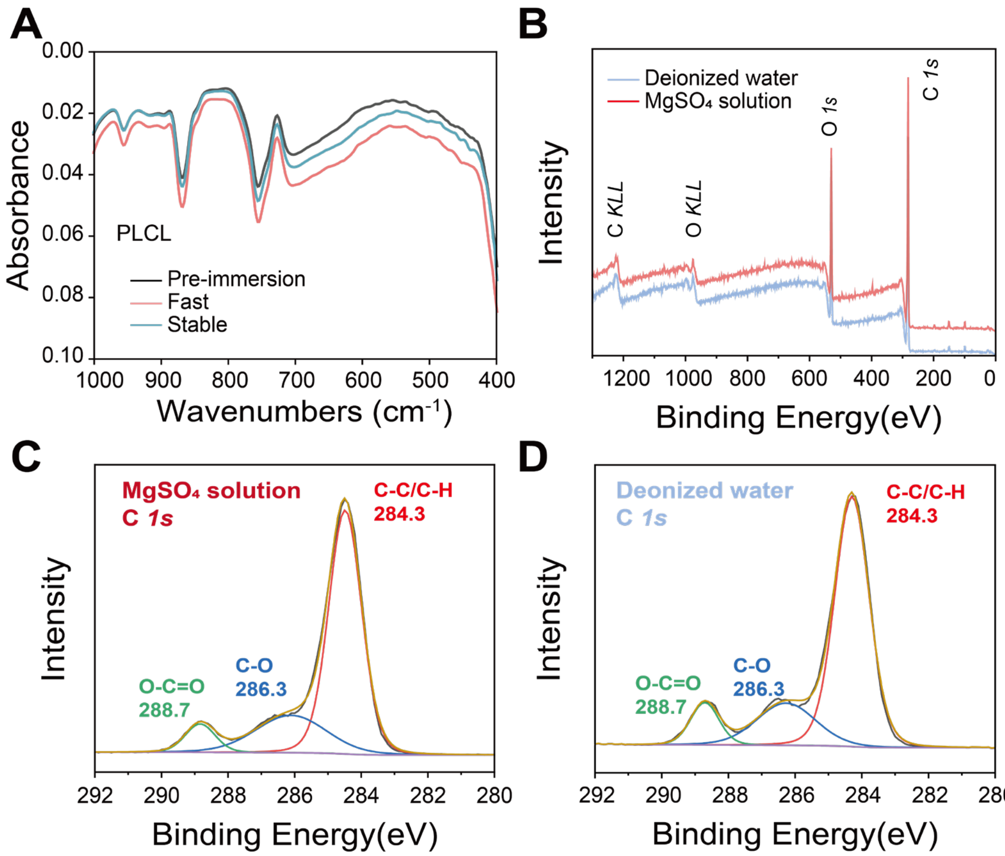

Fig. S8.

(A) FTIR spectra of PLCL stents after immersion for 7 days (Fast) and 56 days (Stable). (B) XPS spectrum of PLCL after immersion in pure water and $MgSO_4$ solution. (D and E) high solution spectrum of C *1s* of PLCL after immersion in pure water and $MgSO_4$ solution.

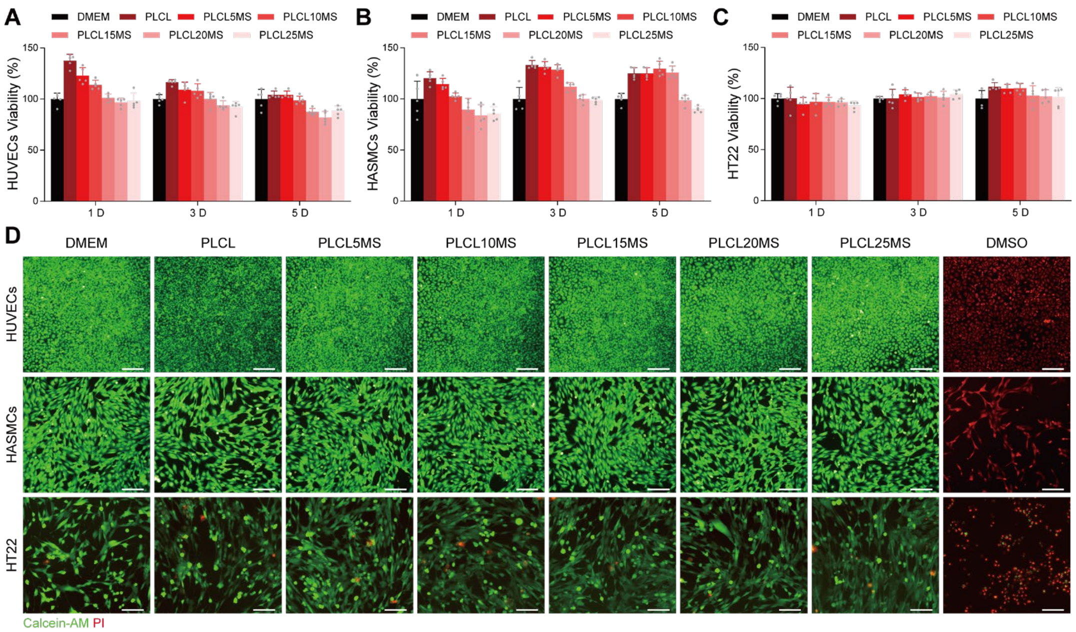

Fig. S9.

In vitro cytotoxicity of PLCL loaded with different concentrations of $MgSO_4$.The cell viability of A) HUVECs, B) HASMCs and C) HT-22 cells were evaluated by CCK-8 assays, respectively. D) Live/dead cell staining (Calcein-AM (green), PI (red)) of HUVECs, HASMCs and HT-22 cells co-cultured with extract of PLCL loaded with different concentrations of $MgSO_4$, scale bars: 20 μm. Source data and exact P values are provided as a Source data file. One-way analysis of variance (ANOVA) with a Tukey/Games-Howell post hoc test for multiple comparisons. Sample size: n=5 biologically independent replicates. Data are presented as mean values ± SD.

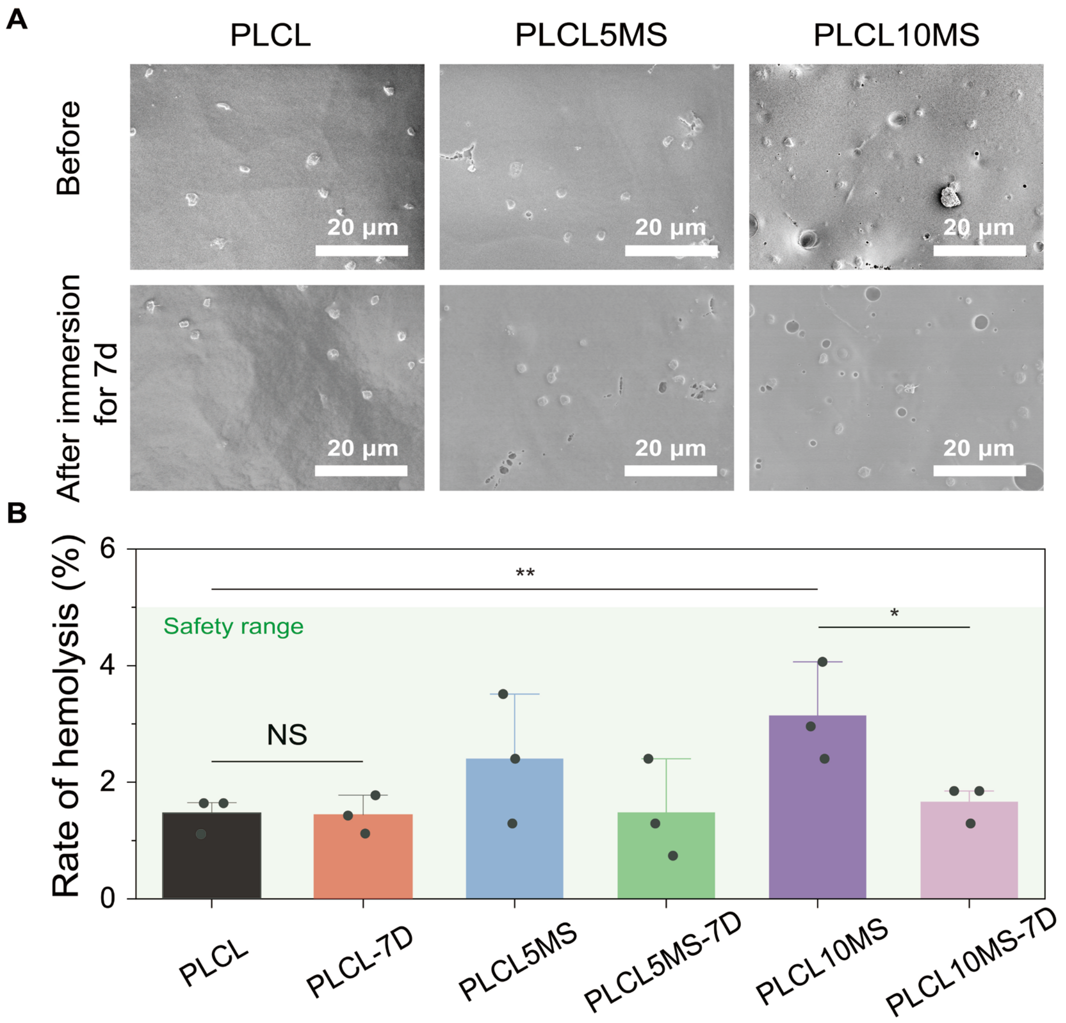

Fig. S10.

(A) Scanning electron microscope images of platelet adhesion on the surface of PLCL, PLCL5MS, and PLCL10MS before and after immersion for 7d; (B) Rate of hemolysis. (n = 3, biologically independent replicates). Source data and exact P values are provided as a Source data file. One-way analysis of variance (ANOVA) with a Tukey/Games-Howell post hoc test for multiple comparisons. * $P< 0.05$, ** $P< 0.01$. Data are presented as mean values ± SD.

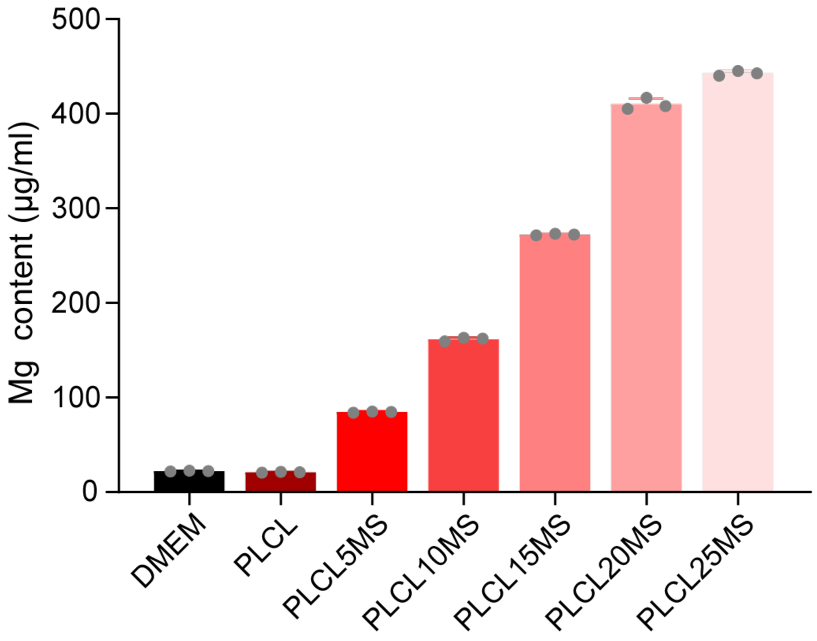

Fig. S11.

Magnesium concentrations in extracts of PLCLxMS composites. Source data and exact P values are provided as a Source data file. One-way analysis of variance (ANOVA) with a Tukey/Games-Howell post hoc test for multiple comparisons. Sample size: n=3 biologically independent replicates. Data are presented as mean values ± SD.

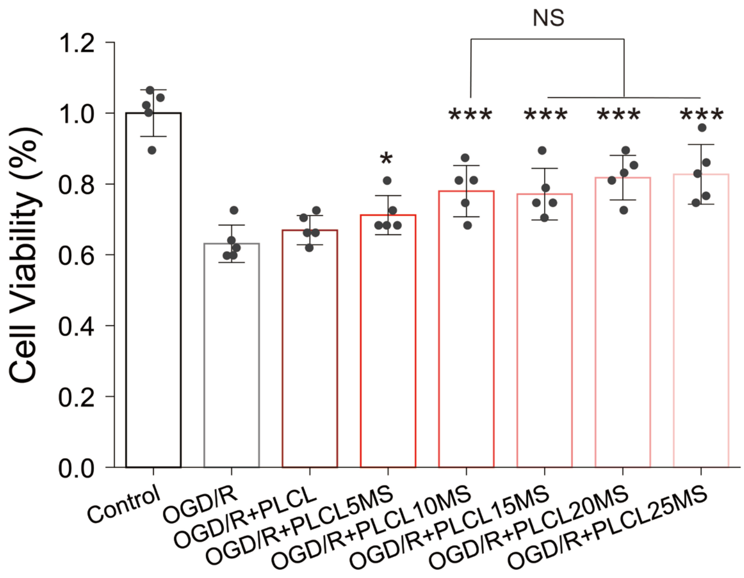

Fig. S12.

In vitro neuroprotection analysis using the OGD/R cell model. The cell viability of SH-SY5Y cells were evaluated by CCK-8 assays. Source data and exact P values are provided as a Source data file. One-way analysis of variance (ANOVA) with a Tukey/Games-Howell post hoc test for multiple comparisons. Sample size: n=5 biologically independent replicates. * $P< 0.05$, *** $P< 0.001$ vs. OGD/R group. NS, not significant. Data are presented as mean values ± SD.

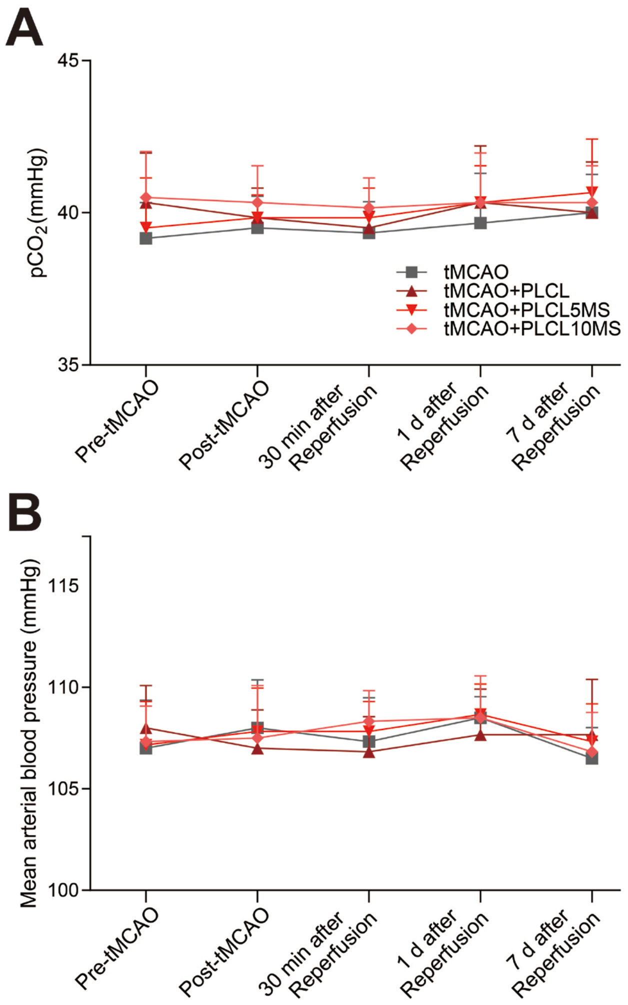

Fig. S13.

Measurement of (A) pCO_2 and (B) mean arterial blood pressure at the end of each experiment of LSI. Source data and exact P values are provided as a Source data file. One-way analysis of variance (ANOVA) with a Tukey/Games-Howell post hoc test for multiple comparisons. Sample size: n=6 biologically independent replicates. Data are presented as mean values ± SD.

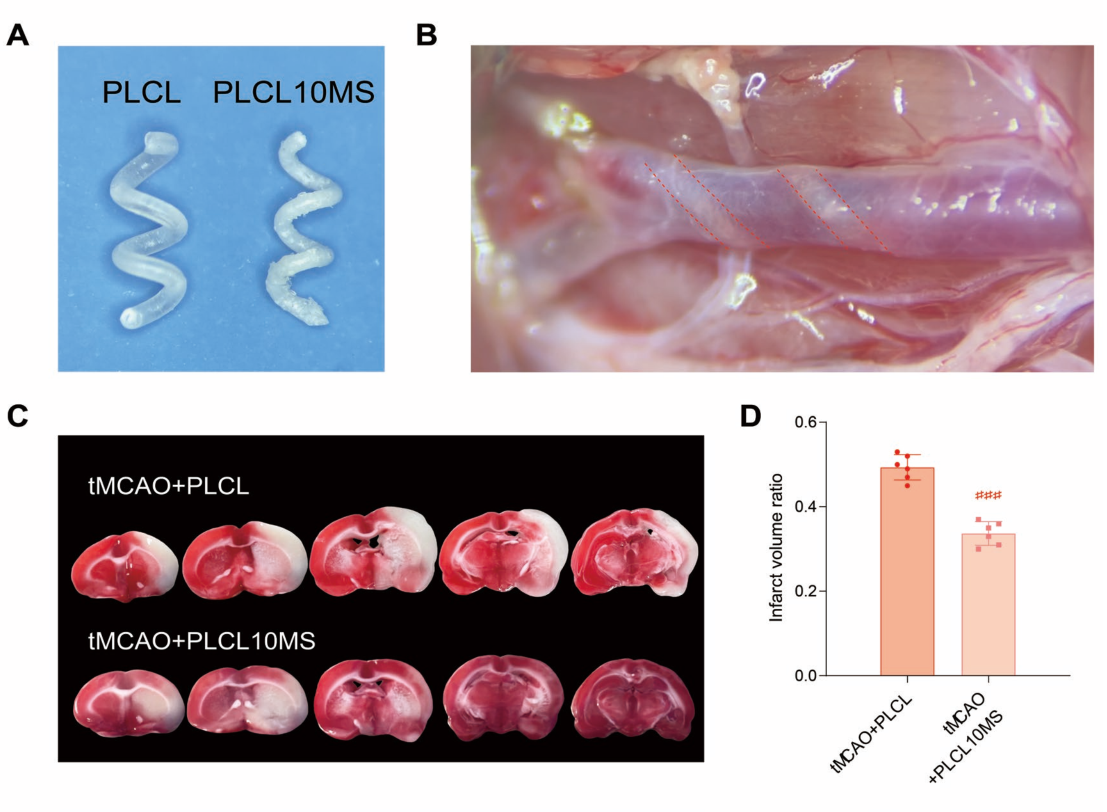

Fig. S14.

(A) Optical images of PLCLxMS spiral stents. (B) Visualization of blood vessel post-stent implantation (red dashed line). (C) Brain slices stained with TTC 1 day after stent implantation. (D) Quantification of brain infarct volume from (C). Source data and exact P values are provided as a Source data file. One-way analysis of variance (ANOVA) with a Tukey/Games-Howell post hoc test for multiple comparisons. Sample size: n=6 biologically independent replicates. ### P< 0.001, MCAO+PLCL10MS vs. MCAO+PLCL group. Data are presented as mean values ± SD.

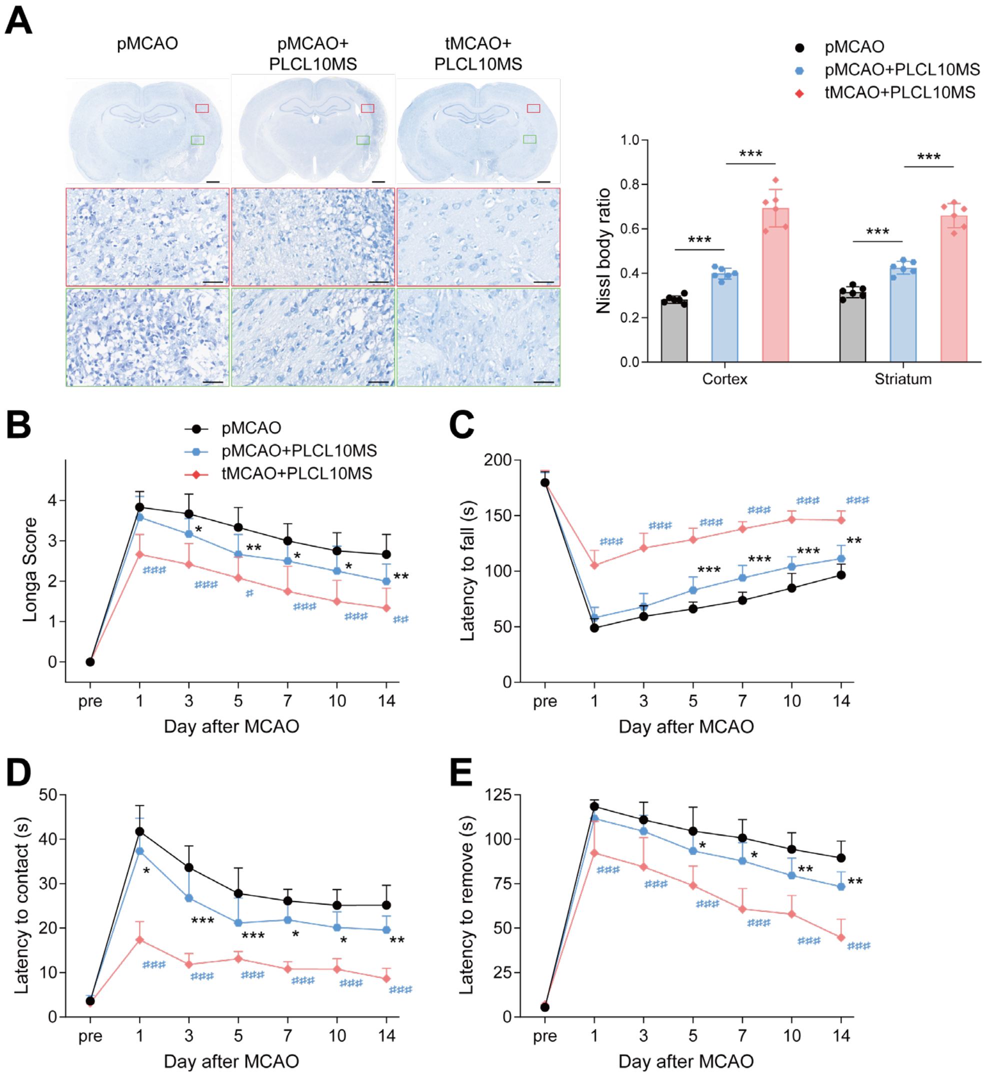

Fig. S15.

Nissl staining and behavioral results of Group pMCAO, Group pMCAO+PLCL10MS and Group tMCAO+PLCL10MS. (A) Brain slices with Nissl staining and quantification of Nissl bodies; scale bars: 1 mm and 25 μm. (B) Results from the Longa score assessments, (C) rotarod test and (D and E) adhesive contact and removal test performance before stroke and up to 14 days post-stroke. Source data and exact P values are provided as a Source data file. One-way analysis of variance (ANOVA) with a Tukey/Games-Howell post hoc test for multiple comparisons. Sample size: n = 6 for nissl staining, biologically independent replicates; n = 12 for neurological tests, biologically independent replicates. * P< 0.05,** P< 0.01, *** P< 0.001, pMCAO+PLCL10MS vs. pMCAO group; # P <0.05, ## P< 0.01, ### P< 0.001, tMCAO+PLCL10MS vs. pMCAO+PLCL10MS group. Data are presented as mean values ± SD.

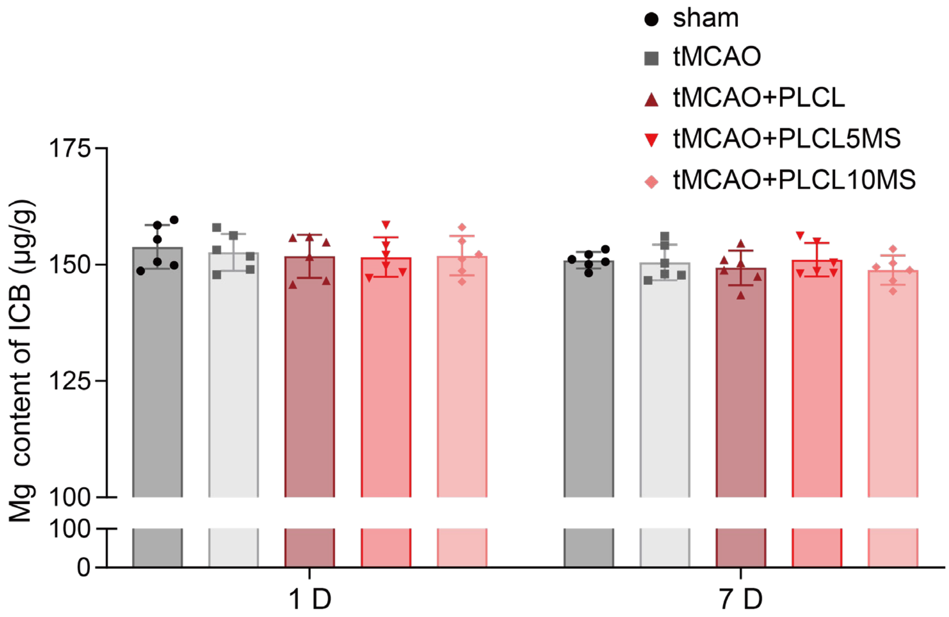

Fig. S16.

Magnesium concentration in the infarction contralateral brain (ICB) of rats at 1 and 7 days post-implantation of PLCLxMS wires. Source data and exact P values are provided as a Source data file. One-way analysis of variance (ANOVA) with a Tukey/Games-Howell post hoc test for multiple comparisons. Sample size: n=6 biologically independent replicates. Data are presented as mean values ± SD.

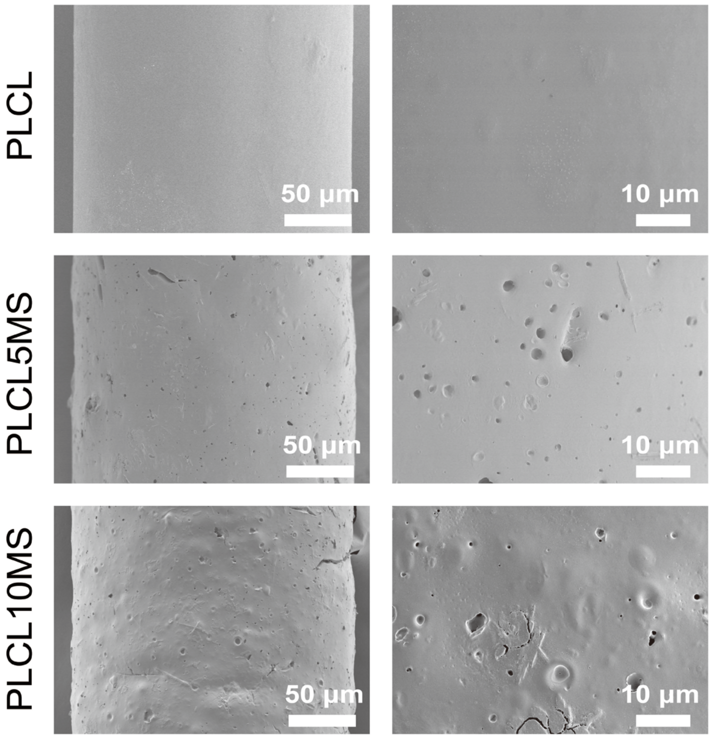

Fig. S17.

Scanning electron microscope images of the PLCL, PLCL5MS and PLCL10MS wires after implantation for 7 days.

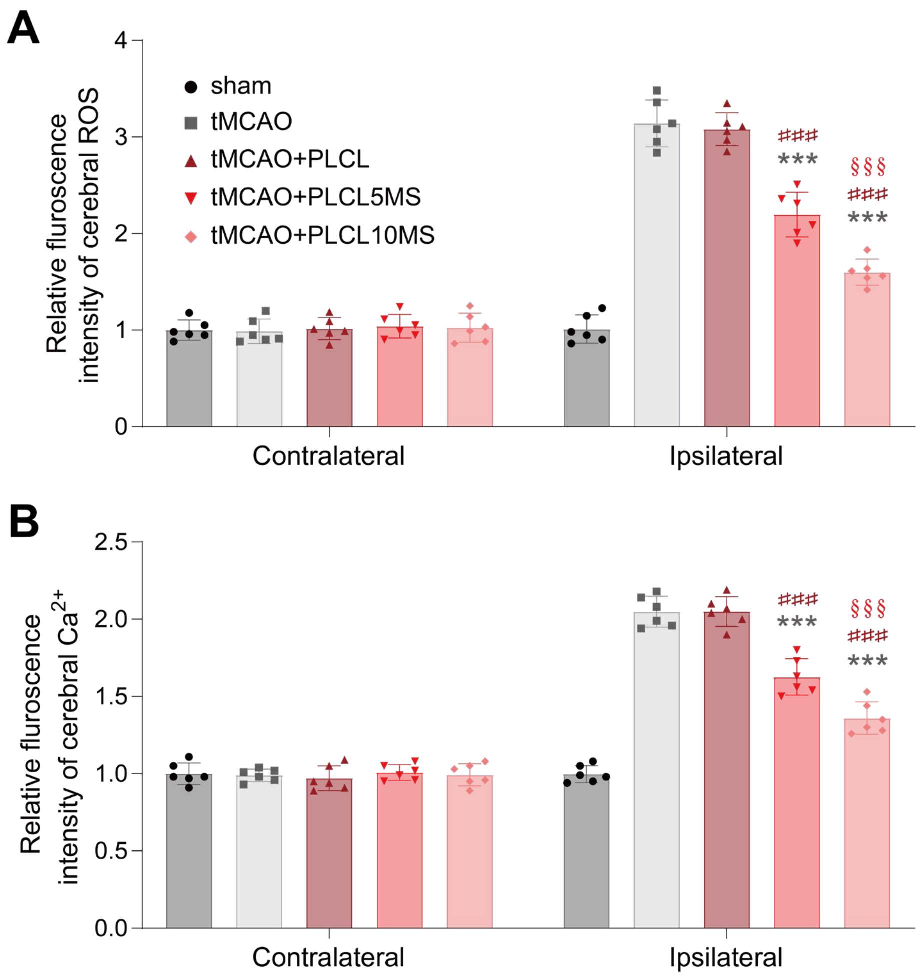

Fig. S18.

Relative cerebral (A) ROS and (B) Ca^{2+} concentration in day 1. Source data and exact P values are provided as a Source data file. One-way analysis of variance (ANOVA) with a Tukey/Games-Howell post hoc test for multiple comparisons. Sample size: n=6 biologically independent replicates. Statistical significance is indicated by *** $p < 0.001$ for tMCAO+PLCL5MS or tMCAO+ PLCL10MS vs. tMCAO; ### $P < 0.001$, tMCAO+PLCL5MS or tMCAO+PLCL10MS vs. tMCAO+PLCL group; §§§ $P < 0.001$, tMCAO+PLCL10MS vs. tMCAO+PLCL5MS. Data are presented as mean values ± SD.

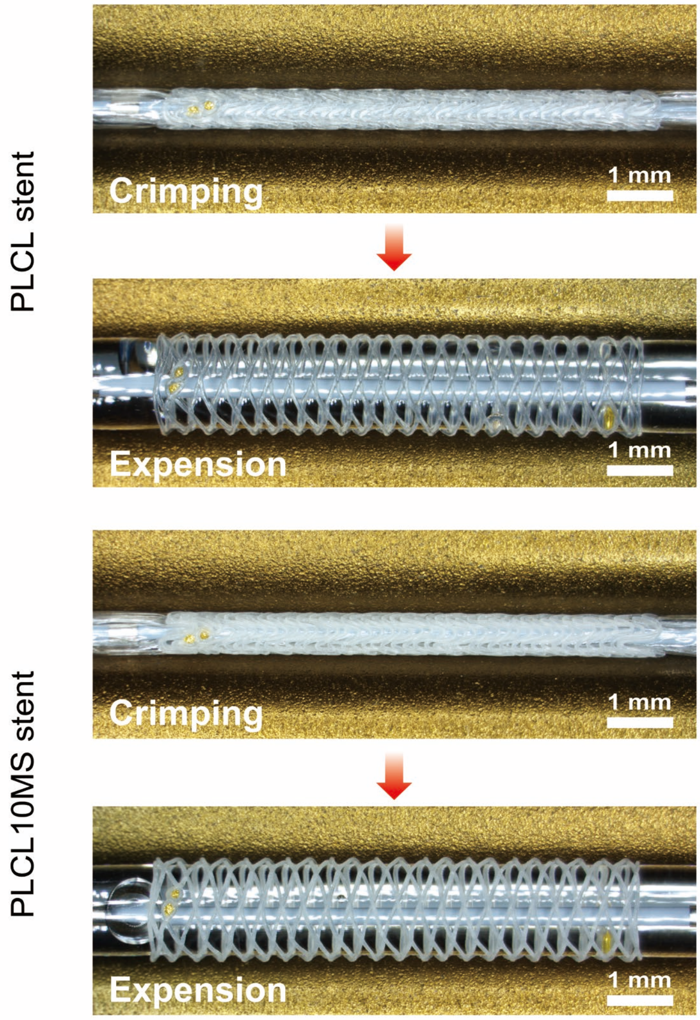

Fig. S19.

Optical images of the PLCL and PLCL10MS stents after crimping and expansion. Source data are provided as a Source Data file.

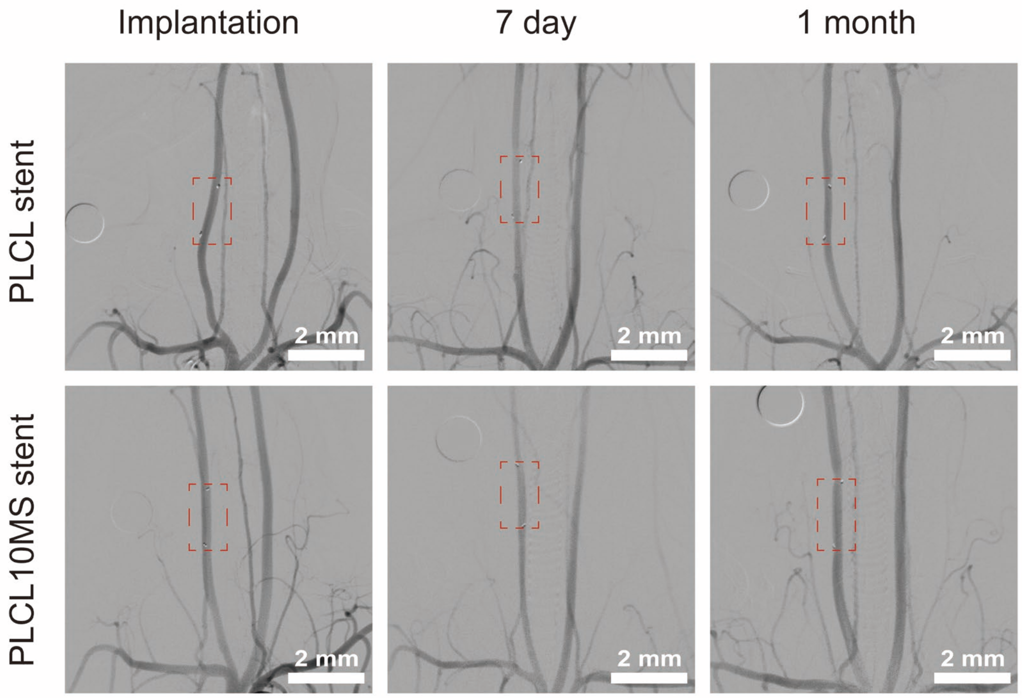

Fig. S20.

DSA immediately, 7 day and 1 month after PLCL and PLCL10MS stents placement. The red dotted box highlights the position of the stents.

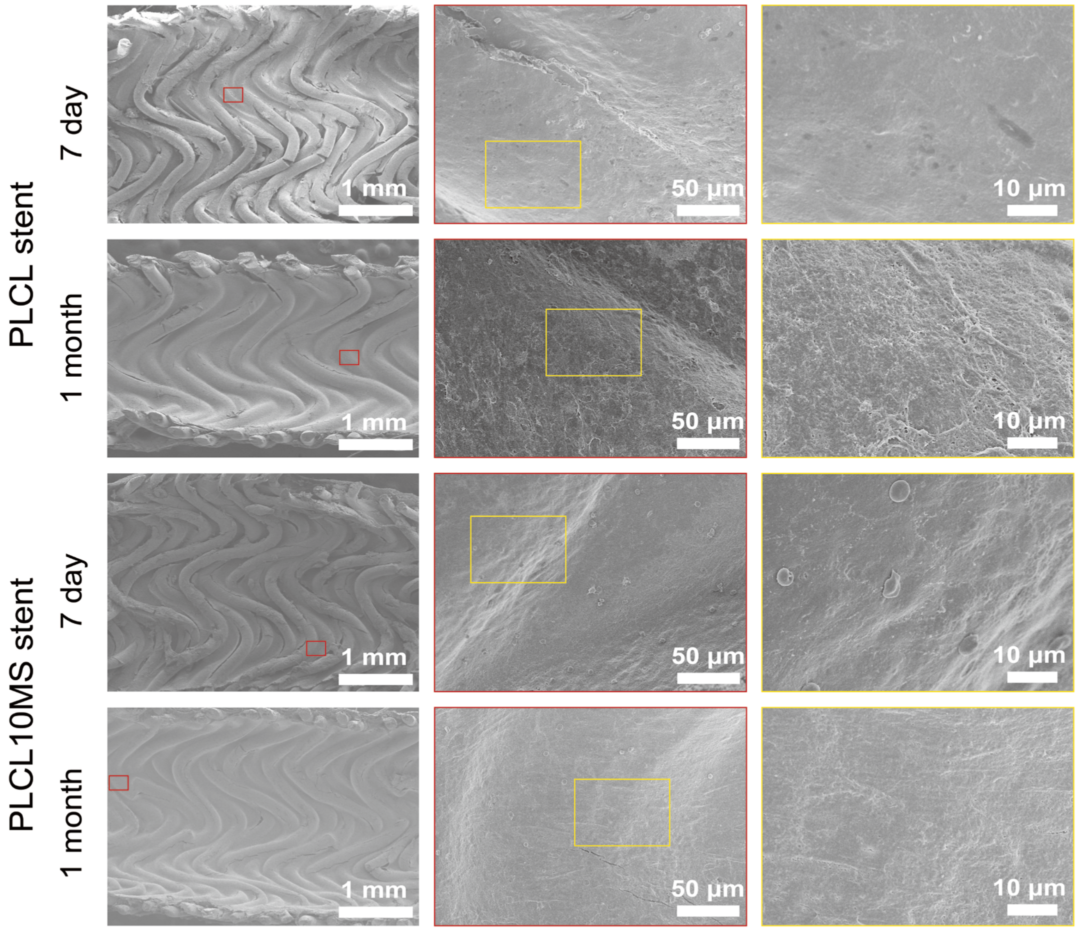

Fig. S21.

Reendothelializations after PLCL and PLCL10MS stents implantation for 7 day and 1 month in rabbit common carotid artery, shown in SEM images. Reendothelializations was completed after 1 months.

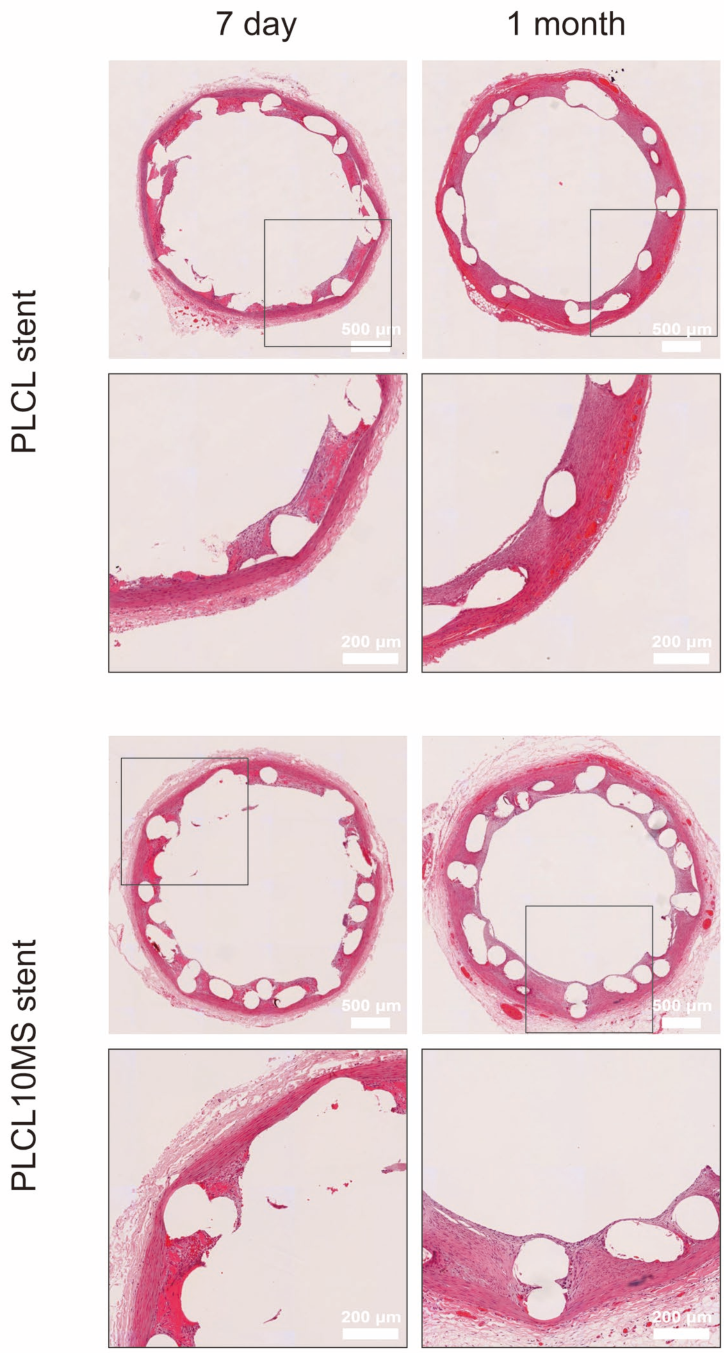

Fig. S22.

Images of PLCL and PLCL10MS stents segments of the common carotid artery stained with HE at 7 day and 1 month post-implantation.

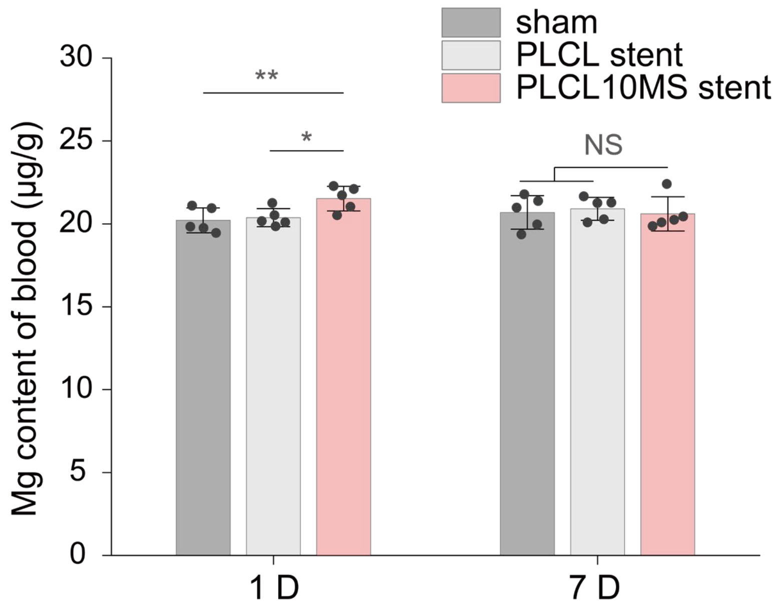

Fig. S23.

Magnesium concentration in the blood of rabbits at 1 and 7 days post-implantation of PLCL and PLCL10MS stents. Source data and exact P values are provided as a Source data file. One-way analysis of variance (ANOVA) with a Tukey/Games-Howell post hoc test for multiple comparisons. Sample size: n = 5 biologically independent replicates. * $P< 0.05$, ** $P< 0.01$. NS, not significant. Data are presented as mean values ± SD.

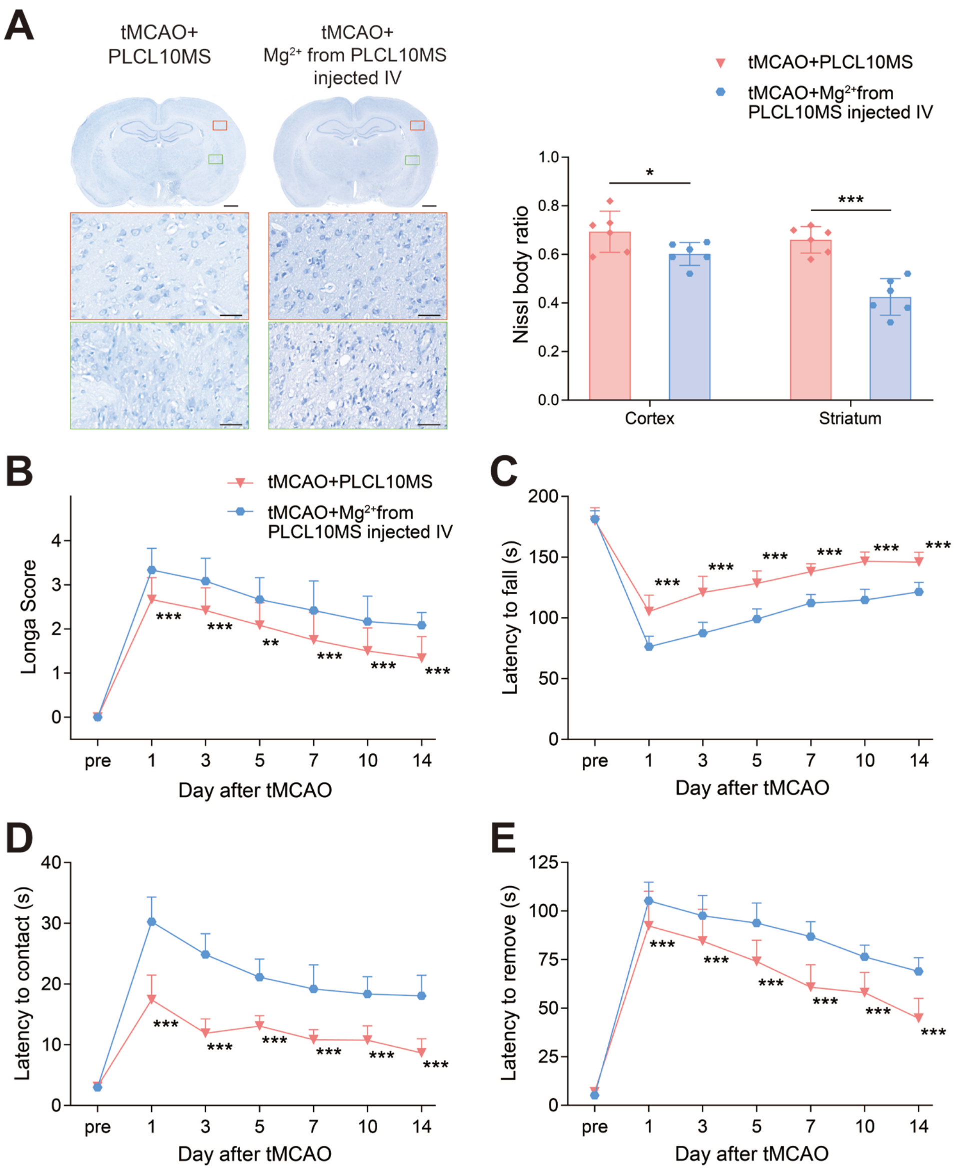

Fig. S24.

Nissl staining and behavioral results of Group tMCAO+Mg^{2+} from PLCL10MS injected IV and Group tMCAO+PLCL10MS. (A) Brain slices with Nissl staining and quantification of Nissl bodies; scale bars: 1mm and 25 µm. (B) Results from the Longa score assessments, (C) rotarod test and (D and E) adhesive contact and removal test performance before stroke and up to 14 days post-stroke. Source data and exact P values are provided as a Source data file. One-way analysis of variance (ANOVA) with a Tukey/Games-Howell post hoc test for multiple comparisons. Sample size: n = 6 for nissl staining, biologically independent replicates; n = 12 for neurological tests, biologically independent replicates. * P< 0.05, ** P< 0.01, *** P< 0.001, tMCAO+PLCL10MS vs. tMCAO+Mg^{2+} from PLCL10MS injected IV group. Data are presented as mean values ± SD.

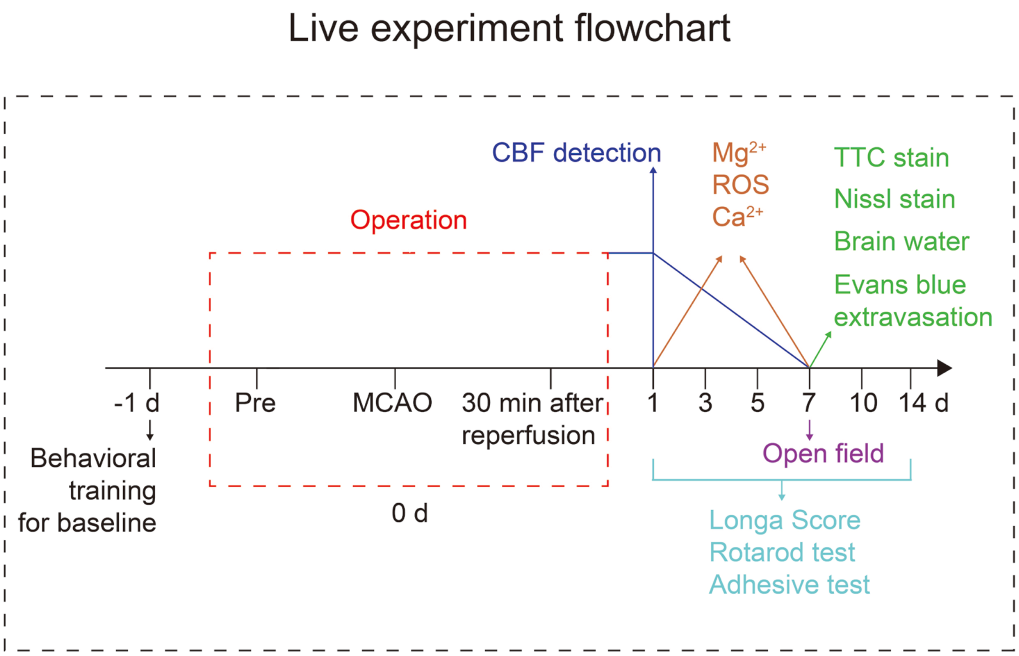

Fig. S25.

Schematic diagram of animal experimental plan.

Table S1. Key characteristics of Staged-Mg^{2+} release

Stage	Time	Rate of Mg^{2+} release ($mg\cdot L^{-1}\cdot day^{-1}$)	Dissolved $MgSO_4$	Mechanism
Burst	Hours	6.49-38.40	Surface	Dissolution
Fast	Weeks	0.15-4.08	Shallow layer	Diffusion
Stable	Months	0.02-0.30	Deep layer	Diffusion

`

Table S2. Typical polymer based stents

Stent	Material	Animal model	Function	Functional Method	Reference
ABSORB BVS	PLLA	Human Coro-nary artery	Antiproliferative	PDLLA coating with Everolimus	2015[63]
DESolve NTx	PLA	Human Coro-nary artery	Anti-proliferative and anti-inflammatory	PLLA coating with Novolimus	2017[64]
MeRes100	PLLA	Human Coro-nary artery	Inhibiting neointima proliferation	PDLLA coating with Sirolimus	2011[65]
XINSORB	PLLA	Porcine coro-nary artery	Inhibiting neointima proliferation	PDLLA mixed with PLLA with Sirolimus	2012[66]
BRS	PLGA	Ovine femoral and profunda vessel	Self-expanding	Composite design: braided stent coated with an elastomer of PGCL and crosslinked	2018[67]
PLCL-$MgSO_4$	PLCL	Rat internal carotid artery	Neuroprotection	Mix $MgSO_4$ with PLCL	This study

PDLLA: Poly(D,L-lactic acid); PBAT: Poly(butyleneadipate-co-terephthalate); PDDA: Poly (dimethyl diallyl ammonium chloride); P(LA-TMC): poly(lactic acid-co-trimethylene carbonate); APTES: 3-amino-propyltrimethoxysilane (APTES); PLGA: poly(lactic-co-glycolic acid); PLLA: Poly-L-lactic Acid; PDLLA: Poly-D-L-Lactic acid; PGCL: poly(glycolide-co-caprolactone);

Table S3. The proposed sequential neuroprotection mechanism of the stent

Stages of ischaemia	Time	Physiological mechanism	Stages of Mg^{2+} release	Neuroprotection of Mg^{2+}	Reference
Hyperacute	Seconds to hours in rodent stroke models	BBB becomes dysregulated; ROS or DNA damage mediated neuronal apoptosis; Ca^{2+} and glutamate mediated excitotoxicity; inflammatory responses mediated secondary injuries	Burst	Inhibit the release of glutamate; inhibit Ca^{2+} overload; improve post-ischemic vascular perfusion; reduce ROS production and mitochondrial damage	[17, 20, 24, 73-75]
Acute	The first 3 weeks of the initial injury in rodent models of stroke	Inflammatory response peaks; the infarct volume ceases to expand; astrocytes proliferate locally to create a physical barrier; pro-repair mechanisms up-regulate;	Fast	Promote proliferation of astrocytes and neurogenesis; reduce ROS production and mitochondrial damage caused by inflammation	[81-83]
Subacute	After Fast stage	Persistent inflammatory responses and new vessels form; Neural circuit plasticity diminish	Stable	Mg^{2+} concentration reaches dynamic equilibrium	[17]

Table S4. Summary of typical studies of biodegradable Mg alloy stents

Material	Implant type	Animal model	Function	Functional Method	Reference
AZ31B	Mg-alloy stents	Porcine coronary arteries	Improving the corrosion resistance	Chemical conversion coating and biodegradable PDLLA coating	2020[91]
AZ31	Mg-alloy stents	NA	Improving the corrosion resistance	PBAT coating	2022 [92]
AZ31	Mg-alloy stents	SD rats abdominal aorta	Anticorrosive and antithrombotic function	PDDA incorporated multilayer coating	2021 [93]
AZ31B	Mg-alloy stents	Rabbit Infrarenal abdominal aorta	Preventing restenosis	P(LA-TMC) coating carrying sirolimus	2011[94]
Mg-2.0Zn-0.5Y-0.5Nd	Mg-alloy stents	Porcine coro-nary artery	Improving corrosion resistance and othelialization	APTES treated silane coating and rapamycin-eluting PLGA layer	2016 [95]
Mg-2.5Nd-0.21Zn-0.44Zr	Mg-alloy stents	Rabbit's abdominal artery	Improving corrosion resistance	Design of the alloy composition	2017 [96]
Mg-2.5Nd-0.21Zn-0.44Zr	Mg-alloy stents	Porcine coronary artery	Improving corrosion resistance and inhibiting neointima proliferation	Rapamycin-eluting PLLA coating and protective MgF_2 layer	2017 [97]
WE43	Mg-alloy stents	Porcine coronary artery	Improving machenical performance	Design of the alloy composition	2008 [98]
DREAMS (WE43)	Mg-alloy stents	Human Coronary artery	Improving corrosion resistance and inhibiting neointima proliferation	PLGA coating with paclitaxel	2013 [99]
DREAMS 2G (WE43)	Mg-alloy stents	Human Coronary artery	Improving corrosion resistance and inhibiting neointima proliferation	PLLA coating with sirolimus	2020 [100]
Mg-8.5Li	Mg-alloy stents	Porcine iliac artery	Improving mechanical performance	Design of the alloy composition	2021 [101]
