# Supplementary material for: Mechanically robust neuroprotective stent by sequential Mg ions release for ischemic stroke therapy
Source: Nat Commun. 2025 Jul 16;16:6557. doi: 10.1038/s41467-025-61199-x (PMC12267439; doi:10.1038/s41467-025-61199-x)
Supplement: Supplementary file 2 — Reporting Summary [file 41467_2025_61199_MOESM2_ESM.pdf]

nature portfolio

Corresponding author(s): Yufeng Zheng

Last updated by author(s): Apr 28, 2025

Reporting Summary

Nature Portfolio wishes to improve the reproducibility of the work that we publish. This form provides structure for consistency and transparency in reporting. For further information on Nature Portfolio policies, see our Editorial Policies and the Editorial Policy Checklist.

Statistics

For all statistical analyses, confirm that the following items are present in the figure legend, table legend, main text, or Methods section.

n/a	Confirmed	
☐	☒	The exact sample size (*n*) for each experimental group/condition, given as a discrete number and unit of measurement
☒	☐	A statement on whether measurements were taken from distinct samples or whether the same sample was measured repeatedly
☐	☒	The statistical test(s) used AND whether they are one- or two-sided<br>*Only common tests should be described solely by name; describe more complex techniques in the Methods section.*
☒	☐	A description of all covariates tested
☒	☐	A description of any assumptions or corrections, such as tests of normality and adjustment for multiple comparisons
☐	☒	A full description of the statistical parameters including central tendency (e.g. means) or other basic estimates (e.g. regression coefficient) AND variation (e.g. standard deviation) or associated estimates of uncertainty (e.g. confidence intervals)
☐	☒	For null hypothesis testing, the test statistic (e.g. *F*, *t*, *r*) with confidence intervals, effect sizes, degrees of freedom and *P* value noted<br>*Give P values as exact values whenever suitable.*
☒	☐	For Bayesian analysis, information on the choice of priors and Markov chain Monte Carlo settings
☒	☐	For hierarchical and complex designs, identification of the appropriate level for tests and full reporting of outcomes
☒	☐	Estimates of effect sizes (e.g. Cohen's *d*, Pearson's *r*), indicating how they were calculated

Our web collection on statistics for biologists contains articles on many of the points above.

Software and code

Policy information about availability of computer code

Data collection	No software was used
Data analysis	SPSS 17.0

For manuscripts utilizing custom algorithms or software that are central to the research but not yet described in published literature, software must be made available to editors and reviewers. We strongly encourage code deposition in a community repository (e.g. GitHub). See the Nature Portfolio guidelines for submitting code & software for further information.

Data

Policy information about availability of data

All manuscripts must include a data availability statement. This statement should provide the following information, where applicable:

- Accession codes, unique identifiers, or web links for publicly available datasets
- A description of any restrictions on data availability
- For clinical datasets or third party data, please ensure that the statement adheres to our policy

The data that support the findings of this paper are available from the corresponding authors upon reasonable request. Correspondence and requests for materials should be addressed to H. Zhang. All the source data related to figures and tables are provided in this paper.

Research involving human participants, their data, or biological material

Policy information about studies with human participants or human data. See also policy information about sex, gender (identity/presentation), and sexual orientation and race, ethnicity and racism.

Reporting on sex and gender	We don't have related issues
Reporting on race, ethnicity, or other socially relevant groupings	We don't have related issues
Population characteristics	We don't have related issues
Recruitment	We don't have related issues
Ethics oversight	We don't have related issues

Note that full information on the approval of the study protocol must also be provided in the manuscript.

Field-specific reporting

Please select the one below that is the best fit for your research. If you are not sure, read the appropriate sections before making your selection.

[x] Life sciences [ ] Behavioural & social sciences [ ] Ecological, evolutionary & environmental sciences

For a reference copy of the document with all sections, see nature.com/documents/nr-reporting-summary-flat.pdf

Life sciences study design

All studies must disclose on these points even when the disclosure is negative.

Sample size	The sample sizes for morphology, microstructure characterization, mechanical evaluation, in vitro degradation test were determined according to statistical requirements.<br>The sample sizes for in vitro biocompatibility test were determined according to the ISO-10993 standard.<br>The sample sizes for MCAO Model and Material Implantation were determined on 3Rs (Replace, Reduce and Refine) principle for animal experiments, and is kept to a minimum number whileobtaining statistically significant data.
Data exclusions	Data were not excluded from analysis.
Replication	All the quantitative data in this study has been repeated for as least 3 times independently. For animal data, as least 5 replications have been performed independently. The n numbers are presented in each figure legend. All attempts at replication were shown with scatter in related figures.
Randomization	The samples in this experiment were randomly assigned, and there was no considered control.
Blinding	The data used for statistics in the article were measured in the same environment and there is no blinding issue.

Reporting for specific materials, systems and methods

We require information from authors about some types of materials, experimental systems and methods used in many studies. Here, indicate whether each material, system or method listed is relevant to your study. If you are not sure if a list item applies to your research, read the appropriate section before selecting a response.

Materials & experimental systems

n/a	Involved in the study	
[x]	[ ]	Antibodies
[ ]	[x]	Eukaryotic cell lines
[x]	[ ]	Palaeontology and archaeology
[ ]	[x]	Animals and other organisms
[x]	[ ]	Clinical data
[x]	[ ]	Dual use research of concern
[x]	[ ]	Plants

Methods

n/a	Involved in the study	
[x]	[ ]	ChIP-seq
[ ]	[x]	Flow cytometry
[x]	[ ]	MRI-based neuroimaging

Eukaryotic cell lines

Policy information about cell lines and Sex and Gender in Research

Cell line source(s)	HUVECs and HASMCs cells were purchased from Wuhan Prucell Life Sciences Co. (Wuhan, China). HT22 and SH-SY5Y were purchased from BeNaCultureCollection (Beijing, China)
Authentication	Commercially purchased cell lines were not authenticated by study participants.
Mycoplasma contamination	All cell lines were tested negative for mycoplasma contamination.
Commonly misidentified lines (See ICLAC register)	No commonly misidentified cell lines were used in the study.

Animals and other research organisms

Policy information about studies involving animals; ARRIVE guidelines recommended for reporting animal research, and Sex and Gender in Research

Laboratory animals	Male Sprague-Dawley (SD) rats weighing between 280 and 320 g were utilized in the investigation. Male New Zealand rabbits weighing between 3 and 3.5 kg were utilized in the investigation
Wild animals	No wild animals were used in the study.
Reporting on sex	The rats and rabbits involved in the animal experiment in this paper are all male, in order to avoid the influence of estrogen of female animal on the experiment.
Field-collected samples	This study did not involve samples collected from the field
Ethics oversight	All experimental procedures received approval from the Capital Medical University's Institutional Animal Investigation Committee and were conducted in accordance with the National Institutes of Health's standards for the Care and Use of Laboratory Animals.

Note that full information on the approval of the study protocol must also be provided in the manuscript.

Plants

Seed stocks	We don't have related issues
Novel plant genotypes	We don't have related issues
Authentication	We don't have related issues

Flow Cytometry

Plots

Confirm that:

- [ ] The axis labels state the marker and fluorochrome used (e.g. CD4-FITC).
- [ ] The axis scales are clearly visible. Include numbers along axes only for bottom left plot of group (a 'group' is an analysis of identical markers).
- [ ] All plots are contour plots with outliers or pseudocolor plots.
- [ ] A numerical value for number of cells or percentage (with statistics) is provided.

Methodology

Sample preparation	*Describe the sample preparation, detailing the biological source of the cells and any tissue processing steps used.*
Instrument	*Identify the instrument used for data collection, specifying make and model number.*
Software	*Describe the software used to collect and analyze the flow cytometry data. For custom code that has been deposited into a community repository, provide accession details.*

Cell population abundance

Describe the abundance of the relevant cell populations within post-sort fractions, providing details on the purity of the samples and how it was determined.

Gating strategy

Describe the gating strategy used for all relevant experiments, specifying the preliminary FSC/SSC gates of the starting cell population, indicating where boundaries between "positive" and "negative" staining cell populations are defined.

☐ Tick this box to confirm that a figure exemplifying the gating strategy is provided in the Supplementary Information.
